# Bacterial profile and antimicrobial susceptibility patterns of isolates among patients diagnosed with surgical site infection at a tertiary teaching hospital in Ethiopia: a prospective cohort study

**DOI:** 10.1186/s12941-021-00440-z

**Published:** 2021-05-10

**Authors:** Gemedo Misha, Legese Chelkeba, Tsegaye Melaku

**Affiliations:** 1 Department of Pharmacy, College of Health Sciences, Arsi University, Assela, Ethiopia; 2 Department of Pharmacology and Clinical Pharmacy, College of Health Sciences, Addis Ababa University, Addis Ababa, Ethiopia; 3 Department of Clinical Pharmacy, School of Pharmacy, Institute of Health, Jimma University, Jimma, Ethiopia; 4 Jimma Medical Center, Institute of Health, Jimma University, Jimma, Ethiopia

**Keywords:** Antimicrobial susceptibility, Bacterial isolates, Surgical site infection, Ethiopia

## Abstract

**Background:**

Globally, surgical site infections are the most reported healthcare-associated infection and common surgical complication. In developing countries such as Ethiopia, there is a paucity of published reports on the microbiologic profile and resistance patterns of an isolates.

**Objective:**

This study aimed at assessing the bacterial profile and antimicrobial susceptibility patterns of isolates among patients diagnosed with surgical site infection at Jimma Medical Center in Ethiopia.

**Methods:**

A prospective cohort study was employed among adult patients who underwent either elective or emergency surgical procedures. All the eligible patients were followed for 30 days for the occurrence of surgical site infection (SSI). From those who developed SSI, infected wound specimens were collected and studied bacteriologically.

**Results:**

Of 251 study participants, 126 (50.2%) of them were females. The mean ± SD age of the patients was 38 ± 16.30 years. The overall postoperative surgical site infection rate was 21.1% and of these 71.7% (38/53) were culture positive. On gram stain analysis, 78% of them were Gram-negative, 11.5% were Gram-positive and 10.5% were a mixture of two microbial growths. *Escherichia coli* accounted for (21.43%), followed by *Pseudomonas aeruginosa* (19.05%), *Proteus species *(spp.) 14.29%), *Staphylococcus aureus* (11.90%), *Klebsiella species* (11.90%), *Citrobacter *spp. (9.5%), *streptococcal* spp. (7.14%), *Coagulase-negative S. aureus *(*CoNS*) (2.38%)

**Conclusion:**

Gram-negative bacteria were the most dominant isolates from surgical sites in the study area. Among the Gram-negative bacilli, *Escherichia coli* were the most common bacteria causing surgical site infection. As there is high antibiotic resistance observed in the current study, it is necessary for routine microbial analysis of samples and their antibiogram.

## Introduction

Infections caused by an invasive surgical procedure that occurs in the wound are commonly referred to as surgical site infections (SSIs) [1]. It is clinically characterized as an infection that occurs within 30 days of surgery (or within a year if an implant is left in place after the procedure) and affects either the incision or deep tissue at the site of the surgery [2]. These infections can be superficial or deep incisional infections, or infections affecting organs or body spaces. SSIs are the most common infections associated with health care settings. They are associated with significant morbidity and over one-third of postoperative deaths have been reported to be linked to SSI [3, 4]. SSI will double the duration of a patient’s hospital stay and therefore increase the cost of health care [4]. Depending on the type of surgery and the severity of the infection, extra costs due to SSI of between 800 and £ 7000 have been recorded [5, 6].

Contamination of wound site and pathogenicity of microorganisms, balanced against the host’s immune response will determine the occurrence of SSI [7–9]. The organism which causes SSI—are usually derived from the endogenous environment, that is the patient skin or opened viscus). Surgical instrument or theatre environment will contaminate the site during operation leads to exogenous causes of SSI [2, 10, 11]. Hematogenous spread of organisms from distant sources of infection can rarely cause SSI by attachment to the prosthesis or other implant left in the operative site. The infection prevention and control practices of SSI are therefore aimed at minimizing the number of pathogens at surgical site [12, 13].

The most common microorganism cultured from SSIs is *Staphylococcus aureus* [14–16]. When a viscus, such as the large bowel, is opened, tissues are likely to be contaminated by numerous organisms. For example, *Enterobacteriaceae* and anaerobes can cause SSI after colorectal surgery [11]. The presence of a foreign body from prosthetic surgery reduces the number of pathogenic organisms required to cause SSI [17, 18]. Microorganisms, which are non-pathogenic such as *Staphylococcus epidermidis*, may also cause SSI in such environment. The type of wound also dictates the presence of microorganisms at surgical sites. For instance, operations on sterile sites have less than 2%, whereas, SSI will occur more than 10% after operations in “contaminated” or “dirty” sites [19, 20].

Resistance patterns of SSI-associated bacteria vary globally, depending on the region, local epidemiology reports, and susceptibility testing methodology. Bacterial resistances pose a challenge and complicated the SSI treatment. Most of the data on drug resistance were obtained from high-income countries [21, 22]. However, there were limited reports on the prevalence and incidence of resistant bacteria causing SSI, especially from developing countries [21, 23]. Therefore, this study aimed at assessing the bacterial profile and antimicrobial susceptibility patterns of isolates among patients diagnosed with surgical site infection at Jimma Medical Center in Ethiopia.

## Methods

### Study area and period

The study was conducted at Jimma Medical Centre (JMC). Jimma Medical Centre is the only teaching and referral hospital in southwest Ethiopia. Geographically, it is located 352 km southwest of Addis Ababa, the capital. JMC provides services for ~ 15,000 inpatients, 160,000 outpatients, 11,000 emergency cases, and 4500 deliveries in a year with a catchment population of over 20 million people. It has around 800 beds with 21 clinical service units. The surgery department has about 286 beds. It has different subunits such as general surgical, gynecology, obstetric/maternity, and orthopedics units. The study was conducted from April 20 to August 20, 2019.

### Study design and population

A prospective cohort study was employed among adult patients (age ≥ 18 years) who underwent either elective or emergency surgical procedures at general surgery, gynecology/obstetric, and orthopedics wards of JMC. All the eligible patients were followed for 30 days for the occurrence of SSI. From those who developed SSI during 30 days of follow up specimen was collected for bacteriological analysis. We excluded patients who were initially diagnosed with SSIs, died within 48 h, or refused to participate in the study.

### Sampling size determination and sampling technique

The sample size was calculated using a single population proportion formula, by considering, 95% confidence level, a 5% margin of error, and a 19.1% estimated proportion of surgical site infections among patients who underwent surgery in Ethiopia [24];

$${\text{n}} = \frac{{({1}.{96})^{{2}} \left( {0.{191}} \right) \, \left( {{1} - 0.{191}} \right) = {239}}}{{0.0{5}^{{2}} }}.$$

Considering 5% non-response rate, the total sample size for this study was **251** patients.

### Data collection procedures and wound swab sample collection

For sociodemographic and clinical characteristics of the patients, predesigned and semi-structured questionnaire was used. The current study used Centers for Disease Control and Prevention (CDC) SSI surveillance methods [25]. Trained study assistants (one nurse and one clinical pharmacist in the profession) conducted indirect surveillance by acquiring patient information using a form containing SSI risk factors.

The clinical evaluation of surgical sites (wound) was done by the attending physician. The clinical features of wound such as pain, redness, swelling, warm skin around the wound, yellow or green discharge, unpleasant odor, fever and chills were considered for clinical diagnosis of surgical site infection. Wounds bed with suspected bacterial infection was prepared for specimen collection by with moist sterile gauze and sterile normal saline. All surgical sites were inspected 24–48 h after surgery at the time of change of dressing. Swabs from wounds were aseptically collected using sterile cotton. For post-discharge surveillance, patients were asked to return for follow-up within 30 days post-discharge at the hospital’s surgical outpatient clinic. If this did not occur, patients were contacted by mobile phone, and, if an SSI was suspected, they were asked to return to JMC to confirm the diagnosis.

### Culture of specimen

The clinical samples (i.e. pus, pus aspirates, and wound swabs) were collected aseptically and processed immediately in the microbiology laboratory within 30 min by placing the swabs into the sterile test tubes having 0.5 mL of sterile normal saline. The collected samples were inoculated onto MacConkeys agar, Blood agar, and chocolate agar plates. Then after, the inoculated MacConkeys and Blood agar plates were incubated in aerobic condition while Chocolate agar plates were incubated in a 5–10% CO_2_ atmosphere environment at 37 °C for 24–48 h.

### Identification of bacterial pathogens

Characterization of cultures was done using morphological appearances on selective and differential media. Based on standard techniques [26] the motility tests and biochemical tests were carried out.

### Antibiotic susceptibility test

From each confirmed culture isolate, a suspension of a pure colony was done in sterile normal saline, which was incubated at 37 °C for at least 15 min. For uniformity of a suspension on Mueller–Hinton agar (Oxoid Ltd) sterile cotton tip applicator stick was used. For antibiotic susceptibility test (AST), the Kirby-Bauer disk diffusion technique was implemented. For the AST different antibiotic disk were used. These were ciprofloxacin (5 μg), penicillin (10 IU), clindamycin (2 μg), gentamycin (10 μg), trimethoprim-sulfamethoxazole (1.25/23.75 μg), erythromycin (15 μg), tetracycline (30 μg), ceftriaxone (30 μg), ampicillin (10 μg), chloramphenicol (30 μg), meropenem (10 μg), ceftazidime (30 μg), vancomycin (30 μg), cefepime(30 μg) and, cefuroxime(30 μg) (Oxoid Ltd). The zone of inhibition was measured using a ruler. The AST result was classified as susceptible, intermediate, and resistant using the Clinical and Laboratory Standards Institute (CLSI) 2018 performance standards for antimicrobial susceptibility testing interpretation [27].

### Data and laboratory quality control

Different techniques were used for data quality management. These included standardization of data collection materials, training of data collectors, and supervision during data collection. To ensure appropriateness of data collection tool, the questionnaire was pretested before the actual study. Quality assurance process that is incorporated in standard operating procedures of the Microbiology Laboratory of JMC was strictly followed for laboratory analysis. An experienced medical laboratory technologist participated in the laboratory identification procedure. The performance of prepared media was checked by inoculating control strains, *S. aureus* (ATCC-25923) and *E. coli* (ATCC-25922) as control. In addition, sterility was checked by incubating 5% of prepared media at 37 °C for 24–48 h, and reagents for gram stain and biochemical tests were cheeked by standard strains of *S. aureus* and *E. coli.*

### Data analysis

Complete data were entered EPI data version 3.1 and exported to statistical package for social science (SPSS) version 22.0. To present antimicrobial susceptibility patterns the descriptive statistics were used. Frequencies and cross-tabulations were used to summarize descriptive statistics. Statistical significance were considered at p values less than or equal to 0.05.

### Ethical consideration

We obtained ethical clearance from institutional review board (IRB) of the institute of health, Jimma University (Reference number: IHRPGD/585/2019). Written informed consent was secured from each study participant. All participants’ information was kept confidential. Patients who developed SSIs were treated according to the protocol of the hospital.

## Results

### Sociodemographic and clinical data

A total of 251 patients were included in the study. Out of 251 patients, 126 (50.2%) were females. The mean ± SD age of the study participant was 38 ± 16.30 years. Nearly three-fourths of patients were from rural areas. More than two-thirds of surgical procedures were emergent. About 148 (59%) of surgical incision sites were abdominal. The clean or clean contaminated dominated the wound class, whereas only 37 (14.74%) patients had contaminated wounds. Nearly one-fourth of patients 61 (24.3%) had an extended duration of preoperative hospital stay of ≥ 7 days (Table 1).

**Table 1:** Sociodemographic and clinical data of study participants

Variables		Total^a^ N (%)	Surgical site infection		${x}^{2}$(p-value)
			Yes [n (%)] 53 (21.1)^a^	No [n (%)] 198 (78.9)^a^	
Sex					
Male		125 (49.80)	34 (64.15)	91 (45.96)	0.019
Female		126 (50.20)	19 (35.85)	107 (54.04)	
Age (years)					
< 60		214 (85.30)	42 (79.2)	172 (86.90)	0.614
≥ 60		37 (14.70)	11 (20.8)	26 (13.10)	
Residence					
Rural		182 (72.50)	10 (18.87)	59 (29.80)	0.113
Urban		69 (27.50)	43 (81.13)	139 (70.20)	
ASA score					
< 3		240 (95.60)	44 (83.02)	196 (99)	< 0.001
≥ 3		11 (4.40)	9 (16.98)	2 (1)	
Comorbidity					
Yes		50 (20)	16 (30.2)	34 (17.20)	0.035
No		201 (80)	37 (69.8)	164 (82.80)	
Preoperative hospital stay					
≤ 7 days		190 (75.70)	43 (81.13)	147 (74.24)	0.299
> 7 days		61 (24.30)	10 (18.87)	51 (25.76)	
Wards					
General surgery		143 (57)	27 (51)	116 (58.60)	< 0.001
Orthopedics		39 (15.50)	19 (35.80)	20 (10.10)	
Gynecology and obsteristics		69 (27.50)	7 (13.20)	62 (31.30)	
Urgency of surgery					
Scheduled		84 (33.50)	8 (15.09)	76 (38.38)	0.001
Emergent		167 (65.50)	45 (84.91)	122 (61.62)	
Duration of surgery					
< 2 h		187 (74.50)	29 (54.72)	158 (79.80)	< 0.001
≥ 2 h		64 (25.50)	24 (45.28)	40 (19.20)	
Types of wound					
Clean or clean contaminated		214 (85.30)	25 (47.17)	189 (95.45)	< 0.001
Contaminated		37 (14.70)	28 (52.83)	9 (4.55)	
Used antibiotic preoperatively		206 (82.10)	50 (94.34)	156 (78.79)	0.009
Used antibiotic post-operatively		45 (17.90)	23 (43.40)	22 (11.11)	< 0.001
Duration of AMP					
Within 24 h		40 (15.94)	3 (5.66)	37 (18.86)	0.007
> 24 h		166 (66.14)	47 (88.68)	119 (60.10)	

*ASA* American Society of Anesthesiologists, *AMP* antimicrobial prophylaxis

^a^Percentages are calculated from the number of a participant in each column

### Disease comorbidity

Fifty (19.92%) of patients were presented with one or more co-morbidities. The common one were cardiac disorder 20 (7.97%), respiratory disorder 7 (2.79%), psychiatry problem 7 (2.79%) diabetic mellitus 6 (2.39%), malignancy 6 (2.39%), and HIV/AIDS 4 (1.59%) (Table 2).

**Table 2:** Prevalence of disease comorbidity among study participants

Variables		Total^a^ N (%)	Surgical site infection		${x}^{2}$(p-value)
			Yes [n (%)] 53^a^	No [n (%)] 198^a^	
Cardiac disorder					
Yes		20 (7.97)	6 (11.32)	14 (7.07)	0.31
No		231 (92.03)	47 (88.68)	184 (92.93)	
Diabetes mellitus					
Yes		6 (2.39)	5 (9.43)	1 (0.51)	< 0.001
No		245 (97.61)	48 (9.57)	197 (99.49)	
Malignancy					
Yes		6 (2.39)	2 (3.77)	4 (2.02)	0.45
No		245 (97.61)	51 (96.23)	194 (97.98)	
HIV/AIDS					
Yes		4 (1.59)	1 (1.89)	3 (1.52)	0.86
No		247 (98.41)	52 (98.11)	195 (98.48)	
Psychiatry problem					
Yes		7 (2.79)	3 (5.66)	4 (2.02)	0.15
No		244 (97.21)	50 (94.34)	194 (97.98)	
Respiratory disorder					
Yes		7 (2.79)	4 (7.55)	3 (1.52)	0.06
No		244 (97.21)	49 (92.45)	195 (98.48)	

*HIV* human immunodeficiency virus, *AIDS* acquired immunodeficiency syndrome

^a^Percentages are calculated from the number of a participant in each column

### Incidence of SSI

**Fig. 1:**
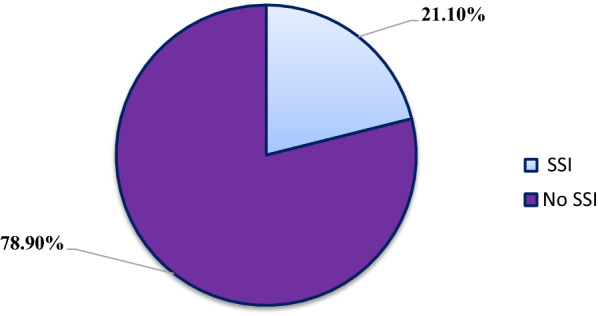
Incidence of surgical site infection among study participants (in percentage)

From a cohort of 251 patients who underwent surgery at Jimma Medical Center, 53 (21.1%) of them developed surgical site infections. Study participants were followed for 6651 person-days. During the study period incidence rate of SSI was 43.74 [95% CI (33.41–57.25)] per 100,000 person-years (Fig. 1).

### Identified bacterial isolates

**Fig. 2:**
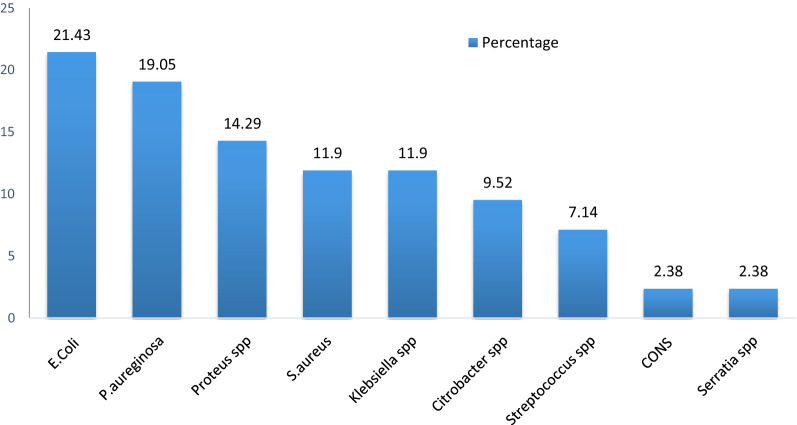
Percentage and types of bacteria among patients diagnosed with surgical site infection

From patients who developed SSI (n = 53), wound swabs or pus aspirates were collected. Out of these, 71.7% (38/53) were culture positive, while the rest, 28.3% (15/53), were culture negative. Out of a total of 38 bacterial isolates, 78% of them were Gram-negative, 11.5% were Gram-positive and 10.5% were a mixture of two microbial growths. Among the types of bacteria identified, *Escherichia coli* accounted for 9 (21.43%), followed by *Pseudomonas aeruginosa* 8 (19.05%), *Proteus species *spp. 6 (14.29%), *Staphylococcus aureus* 5 (11.90%), *Klebsiella species* 5 (11.90%), *Citrobacter *spp.* 4 *(9.5%), *Streptococcal *spp. 3 (7.14%), *Coagulase-negative S. aureus (CoNS)* 1 (2.38%) and *Serratia *spp. 1 (2.38%) (Fig. 2).

### Antibiotic susceptibility pattern of SSI isolates

Antibiotic resistance profiles were reported for the organisms isolated from surgical incision site of infected patients. The Gram-positive pathogens showed high resistance toward penicillin (66.67%), erythromycin (66.67%), and clindamycin (66.67%). The Gram-negative pathogens showed high resistance toward Cefepime (87.88%), ceftriaxone (78.79%), Cefuroxime (63.63%), cotrimoxazole (54.55%), ciprofloxacin (60.60%), and ampicillin (60.60%). Clindamycin and erythromycin-resistant *S. aureus* accounted 80% of all *S. aureus* isolates and showed resistance toward cotrimoxazole (60%). However, only one strain of it showed resistance to vancomycin. Two of three isolates of *streptococcus* were resistant to Penicillin, erythromycin, vancomycin, and clindamycin. All strains of *Pseudomonas aeruginosa* (*P. aeruginosa*) and *proteus *spp. were resistant to Ceftriaxone. *Pseudomonas aeruginosa* isolates were resistant to meropenem (62.5%), ceftazidime (62.5%) ciprofloxacin (50%), and gentamicin (50%). All strains of *Proteus *spp. showed were resistant to cefepime and cefuroxime. Similarly, all isolates of *Citrobacter *spp. showed resistance to cefepime. The identified Serratia spp. were resistant to all tested antibiotics. Meropenem is 100% effective against *E. coli* which was the predominant pathogen in this study (Table 3).

**Table 3:** Antibiotic resistance profile of bacteria isolates from surgical sites

S. no.	Antibiotics tested	Gram-positives			Gram-negatives						Total
		*S. aureus* (n = 5)	*CoNS* (n = 1)	*Streptococcus* (n = 3)	*P. aeruginosa* (n = 8)	*Proteus *spp. (n = 6)	*E. Coli *(n = 9)	*Klebsiella *spp. (n = 5)	*Citrobacter *spp. (n = 4)	*Serratia *spp. (n = 1)	
		Resistance; n	Resistance; n	Resistance; n	Resistance; n	Resistance; n	Resistance; n	Resistance; n	Resistance; n	Resistance; n	Resistance n (%)
1	Penicillin	3	1	2	ND	ND	ND	ND	ND	ND	6 (66.7)
2	Erythromycin	4	Ds	2	ND	ND	ND	ND	ND	ND	6 (66.7)
3	Vancomycin	1	Ds	2	ND	ND	ND	ND	ND	ND	3 (33.3)
4	Clindamycin	4	Ds	2	ND	ND	ND	ND	ND	ND	6 (66.7)
5	Tetracycline	Ds	Ds	1	ND	ND	ND	ND	ND	ND	1 (11.1)
6	Cotrimoxazole	3	Ds	1	6	4	2	2	3	1	22 (52.4)
7	Chloramphenicol	Ds	Ds	1	4	4	1	4	2	ND	16 (48.48)
8	Ampicillin	ND	ND	ND	3	4	4	5	3	1	20 (60.60)
9	Cefepime	ND	ND	ND	8	6	6	4	4	1	29 (87.88)
10	Ceftazidime	ND	ND	ND	5	2	4	2	3	1	17 (51.51)
11	Ceftriaxone	ND	ND	ND	8	6	5	3	3	1	26 (78.79)
12	Cefuroxime	ND	ND	ND	6	6	5	3	1	ND	21 (63.63)
13	Ciprofloxacin	ND	ND	ND	4	4	5	4	3	ND	20 (60.60)
14	Meropenem	ND	ND	ND	5	3	Ds	2	1	1	12 (36.36)
15	Gentamycin	ND	ND	ND	4	2	3	2	1	1	13 (39.39)

Percentage of resistance strain is calculated by dividing of the number of resistance specific isolate to the identified isolate (e.g. there were 5 isolates of *S. aureus* identified; of which 4 are resistance to Erythromycin, which showed the Erythromycin of 80% (4/5)

*ND* not done, *Ds* done susceptible, *CONS* coagulase-negative staphylococcus

## Discussion

Postoperative SSI remains one of the most significant causes of morbidity among surgically treated patients. These patients incur higher costs due to longer hospitalizations, more nursing care, additional wound care, potential hospital admissions, and further surgical procedures. Identification of bacterial pathogens and the selection of an effective antibiotic against the organism are essential in successful management of bacterial infection. In the current study, the overall culture positivity rate from patients with surgical site infection was 71.7%, which was slightly higher than results previously reported from India (68%) [28], but lower than a report from Mekelle (75%) [29] and Nigeria (82%) [30]. Lower rates of positive culture were reported from India (50%) [31] and Nepal (63.3%) [16], Bangladesh (61.8%) [32].

The isolation rate of Gram-negative bacteria was greater (78%) than Gram-positive bacteria (11.5%) in this study. This, in contrast, to study done from Bangladesh [32], and Nepal [33, 34]. This might be related to the study population. In the latter studies, most of the patients were from the orthopedics department where Gram-positive bacteria such as s*taphylococcus* and *streptococcus* are the main causative agents [35, 36]. The prevalence of mixed infections in the current study (10.5%) was lower than previous study from Jimma (22.9%) [37], Dessie (18.5%) [38] and Nigeria (33.2%) [39]. The difference might be due to difference in identification methods, which will influence is known to influence the relative prevalence of bacteria.

Similar to the present study, S.M. Patel et al. [40] demonstrated that *Escherichia coli* (35.7%)was the most common pathogenic isolate followed by *Staphylococcus aureus* (21.4%), Pseudomonas aeruginosa (14.3%), and Klebsiella spp. (14.3%). In a similar study from India [41] and Chennai [11] *Escherichia coli* (41.17%) was reported as the most common bacterial isolates, followed by *Staphylococcus aureus* (13.72%), *Klebsiella pneumonia *(9.80%), *Pseudomonas aeruginosa* (7.84%). Varsha Shahane et al. [42] has also demonstrated *Escherichia coli* as the commonest isolate in their studies. A similar study finding from Gondar (Ethiopia) [43] reported that *Escherichia coli* a major isolate. However, this finding was in contrast with many other studies [29, 35, 44–49]. In these studies, authors have observed *Staphylococcus aureus* as the commonest pathogen causing SSI in their respective studies. The difference in the report might be explained by the difference in the setting and study population. In the current study, most of the patients were from the general surgery ward. Most of the surgical procedures performed were laparotomies and most wounds were either clean-contaminated or contaminated, which had spillage from the gastrointestinal tract. This might be due *to Escherichia coli’s* natural habitat is the gastrointestinal tract. In the current study, a relatively low orthopedic procedure was done, where *staphylococcus* predominates as the causative agent.

In this study, *Proteus *spp. conferred high resistant to Cefepime (100%), Cefuroxime (100%) ceftriaxone (100%), ciprofloxacin (67%), ampicillin (67%), cotrimoxazole (67%) and chloramphenicol (67%) which agrees reports in other studies [37, 38]. In this study, multi-drug resistance (MDR) to commonly used antibiotics was identified. Resistance to antibiotics ranged from 11.1% to 100%. Similarly, a study from Mekelle (Ethiopia) [29] showed a multi-drug resistance to the commonly used antibiotics. In Tikur Anbessa specialized Hospital [50], about 95% of the isolates were resistance to two or more antimicrobials while 82.3% of them were resistance to three or more antimicrobials. Besides these similar national studies, the current study findings were consistent with many other global studies [4, 13, 29, 43, 45, 50, 51]. This might be because these antibiotics are widely prescribed empirically for the treatment of various infections in our setting.

Overall, ceftriaxone resistance in this study was about 78.79%. All *pseudomonas* and *Proteus *spp. isolated were 100% resistant to ceftriaxone. This remarkably higher resistance might be the indiscriminate use of ceftriaxone as prophylaxis to all who underwent surgery in our hospital. Even though, high drug resistance was observed by this study meropenem was effective against *Escherichia coli* which was the predominant cause of SSI in the current study. A high rate (nearly half) of bacterial resistance against chloramphenicol and cotrimoxazole was observed. This is consistent with a study done in Saudi Arabia [49]. This might be due to the indiscriminate use of antibiotics in both hospitals.

In this study, *Citrobacter* maximum resistance was conferred to cefepime (100%), ampicillin (75%), ciprofloxacin (75%), ceftazidime (75%), cotrimoxazole (75%) and ceftriaxone (75%), which was comparable to the result reported Girma et al. [37] in contrast another study report 66.7% resistance for ampicilin [52]. The difference might be due to the setting and included patients in the study. The consumption of cefepime in the study setting is very low. Thus, the high resistance of Citrobacter to such antibiotics needs special attention, especially on their empirical use. Only one-third of *streptococcus *spp. isolates were sensitive to penicillin, erythromycin, vancomycin, and clindamycin. This showed great concern for an infectious condition caused by these bacteria species such as pneumonia, meningitis. The rise in antibiotic resistance emphasizes the importance of sound hospital infection control, rational prescribing policies, and the need for new antimicrobial drugs and vaccines. In general, the current study showed that the reported antibiotic susceptibility data was similar to the previous overall susceptibility pattern of isolates in the study area [53–55]. However, some of the virulent bacteria such as *P. aeruginosa, E. coli, and S. aureus* showed increasing trends in resistance [56–58].

### Limitations of the study

It was not possible to include anaerobic bacteria due to the lack of microbiology laboratory facilities constraints during the study period.

## Conclusion

In conclusion, Gram-negative bacteria were the most dominant isolates from surgical sites in the study area. Among the Gram-negative bacilli, *Escherichia coli* were the most common bacteria causing surgical site infection. A multi-center study should be conducted to see the actual incidence of resistance isolates among patients with wound infection. As there is high antibiotic resistance observed in the current study, it is necessary for routine microbial analysis of samples and their antibiogram. In addition, we recommend proper infection prevention practices to break the disease transmission cycle, strengthening the available antimicrobial stewardship program in the setting and periodic antimicrobial surveillance.

## Data Availability

The data sets generated during and/or analyzed during the current study are available from the corresponding authors on reasonable request.

## Abbreviations

CoNS: Coagulase negative *S. aureus*
CLSI: Clinical and Laboratory Standards Institute
SSIs: Surgical site infections
JMC: Jimma Medical Centre
MDR: Multi-drug resistances
